# Transapical transcatheter mitral valve implantation in a patient with mitral annular defect due to a patch dehiscence after aortic valve replacement: a case report

**DOI:** 10.1093/ehjcr/ytag441

**Published:** 2026-06-12

**Authors:** Vanessa Justine Trenkpohl, Mariia Emelianova, Felix Rudolph, Muhammed Gerçek, Kai Friedrichs

**Affiliations:** Department of General and Interventional Cardiology/Angiology, Heart and Diabetes Center NRW, Ruhr-University Bochum, Bad Oeynhausen 32545, Germany; Department of General and Interventional Cardiology/Angiology, Heart and Diabetes Center NRW, Faculty of Medicine, Bielefeld University, Bad Oeynhausen 32545, Germany; Department of General and Interventional Cardiology/Angiology, Heart and Diabetes Center NRW, Ruhr-University Bochum, Bad Oeynhausen 32545, Germany; Department of General and Interventional Cardiology/Angiology, Heart and Diabetes Center NRW, Faculty of Medicine, Bielefeld University, Bad Oeynhausen 32545, Germany; Department of General and Interventional Cardiology/Angiology, Heart and Diabetes Center NRW, Ruhr-University Bochum, Bad Oeynhausen 32545, Germany; Department of General and Interventional Cardiology/Angiology, Heart and Diabetes Center NRW, Faculty of Medicine, Bielefeld University, Bad Oeynhausen 32545, Germany; Department of General and Interventional Cardiology/Angiology, Heart and Diabetes Center NRW, Ruhr-University Bochum, Bad Oeynhausen 32545, Germany; Department of General and Interventional Cardiology/Angiology, Heart and Diabetes Center NRW, Faculty of Medicine, Bielefeld University, Bad Oeynhausen 32545, Germany; Department of General and Interventional Cardiology/Angiology, Heart and Diabetes Center NRW, Ruhr-University Bochum, Bad Oeynhausen 32545, Germany; Department of General and Interventional Cardiology/Angiology, Heart and Diabetes Center NRW, Faculty of Medicine, Bielefeld University, Bad Oeynhausen 32545, Germany

**Keywords:** Transapical mitral valve implantation, TMVI, Tendyne, Mechanical aortic valve prosthesis, Leaflet perforation, Patch dehiscence, High-risk patient, Transcatheter edge-to-edge repair, TEER, Case report

## Abstract

**Background:**

Transcatheter mitral valve implantation (TMVI) has emerged as a therapeutic option for high-risk patients with severe mitral regurgitation (MR) who are unsuitable for surgery. The Tendyne system, with its dual-frame design consisting of an atrial cuff and an external frame, has shown promising in complex anatomical settings. However, experience with Tendyne TMVI remains limited in patients with post-surgical MR caused by mitral leaflet perforation and a concomitant mechanical aortic valve prosthesis.

**Case summary:**

An 80-year-old woman with a prior mechanical aortic valve replacement (St. Jude Medical, 21 mm) for bicuspid aortic stenosis presented with severe mixed MR. At the index operation, a Manouguian aortic root enlargement using a pericardial patch had been performed to prevent prosthesis-patient mismatch. During follow-up, patch dehiscence resulted in perforation of the anterior mitral leaflet, while concomitant annular dilatation generated an additional regurgitant jet, resulting in severe mixed MR. Given the prohibitive risk of redo cardiac surgery (EuroSCORE II 25.24%), the multidisciplinary Heart Team recommended a transcatheter treatment strategy. Following Heart Team evaluation, transapical TMVI with a Tendyne prosthesis (29S LP) was performed. The procedure achieved complete elimination of MR without residual paravalvular leak or interference with the mechanical aortic valve. Notably, the external stent frame of the prosthesis successfully sealed the mitral leaflet perforation, eliminating the need for additional closure devices.

**Discussion:**

This case underscores the importance of imaging-guided, multidisciplinary Heart Team planning in optimizing procedural outcomes for patients with challenging post-surgical anatomy. Transcatheter mitral valve implantation might be a feasible option for patients with MR after cardiac surgery at prohibitive surgical risk.

Learning points

- Severe mitral regurgitation after aortic root enlargement and mechanical aortic valve replacement poses major surgical and interventional challenges.
- Transcatheter edge-to-edge repair may be unsuitable in the presence of leaflet perforation or paravalvular leakage.
- The dual-frame design of the Tendyne® transcatheter mitral valve implantation prosthesis can effectively seal complex mitral defects without interference with existing mechanical valves.
- Pre-procedural imaging and Heart Team decision-making are key to selecting the optimal transcatheter strategy.

## Introduction

The management of severe mitral regurgitation (MR) in patients with a pre-existing mechanical aortic valve prosthesis presents a complex clinical challenge, particularly in elderly individuals with multiple comorbidities. Redo surgical valve replacement in this population carries a substantial procedural risk, and mitral transcatheter edge-to-edge repair (M-TEER) is anatomically unsuitable in the presence of leaflet perforation or paravalvular defects.^1^

Transapical transcatheter mitral valve implantation (TMVI) with the Tendyne® bioprosthesis has emerged as an innovative therapeutic option for high-risk patients in whom both surgical reoperation and M-TEER are not feasible.^2,3^ The Tendyne prosthesis incorporates an atrial cuff and an external stent frame, which may provide effective sealing of paravalvular or leaflet defects, potentially addressing highly complex anatomical situations.

We present the first documented case in which the external stent frame of the Tendyne prosthesis successfully sealed a leaflet perforation caused by patch dehiscence in a patient with prior mechanical aortic valve replacement with concomitant aortic root enlargement, with no adverse interaction between the two prosthetic valves.

## Summary figure

**Figure:**
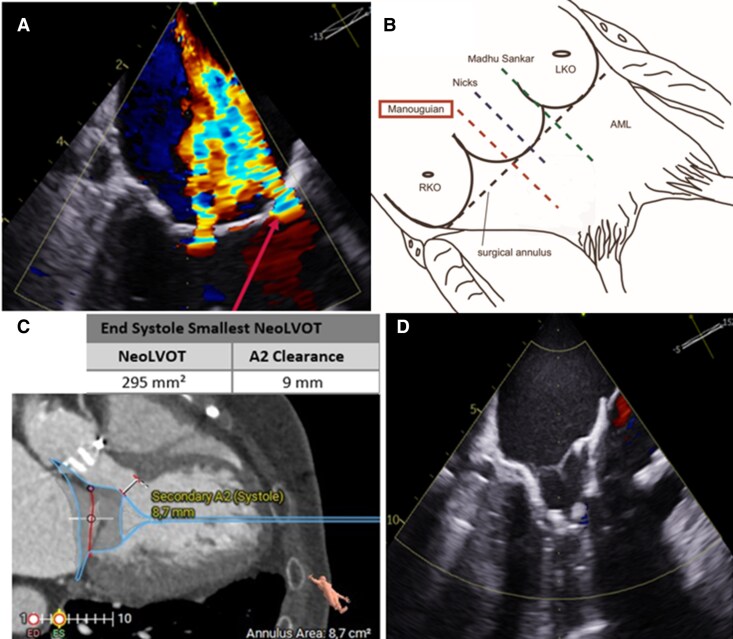
Panel (*A*) depicts mixed severe mitral regurgitation resulting from annular dilatation and leaflet perforation, with the arrow indicating a perforation secondary to patch dehiscence. Panel (*B*) illustrates various surgical techniques for aortic root enlargement, with the Manouguian technique highlighted in red. Panel (*C*) shows computed tomography (CT)–based measurement of the Tendyne device in the setting of a mechanical aortic valve prosthesis, demonstrating an end-systolic neo-left ventricular outflow tract (neo-LVOT) area of 295 mm^2^. Panel (*D*) presents the post-procedural result, with no residual regurgitation detectable.

## Case presentation

An 80-year-old female patient presented with progressively worsening dyspnoea, corresponding to New York Heart Association (NYHA) functional class III. Her medical history was notable for mechanical aortic valve replacement (St. Jude Medical, 21 mm) performed in September 2009 for bicuspid aortic valve stenosis, combined with an aortic root enlargement using the Manouguian technique to prevent patient–prosthesis mismatch^4^ (*Figure 1*). The enlargement was achieved with a patch, which subsequently developed dehiscence (*Figure 2*).

**Figure 1:**
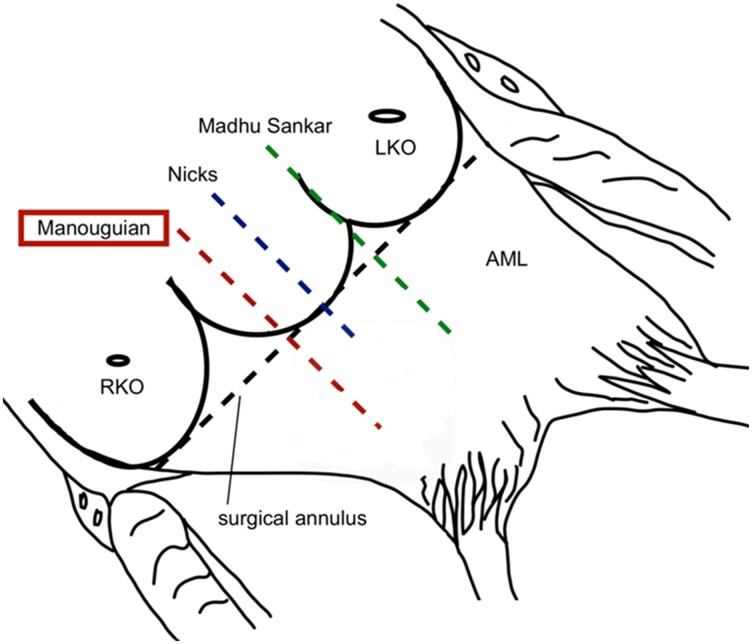
Schematic illustration of surgical techniques for aortic root enlargement to prevent patient–prosthesis mismatch. The Manouguian technique is highlighted in red. Illustration created by the authors.

**Figure 2:**
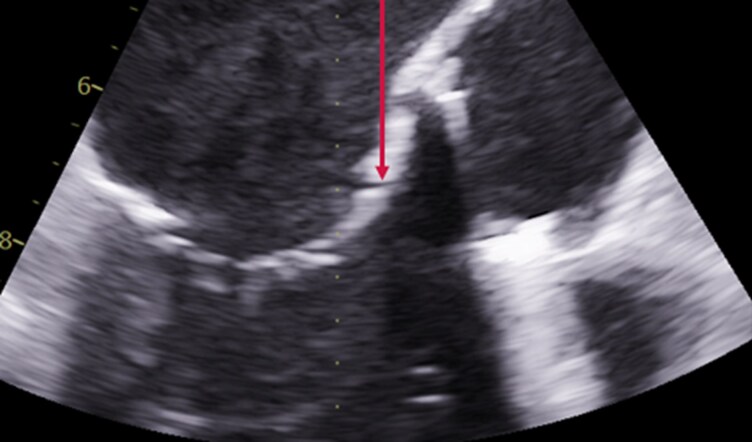
Transoesophageal echocardiography demonstrated a regurgitant jet at the anterior mitral leaflet due to patch dehiscence. The red arrow indicates the site of leaflet perforation.

Laboratory investigations demonstrated a serum creatinine of 1.6 mg/dL with an estimated glomerular filtration rate of 31 mL/min/1.73 m^2^, a haemoglobin level of 13 g/dL, and an N-terminal pro–B-type natriuretic peptide concentration (NT-proBNP) of 3280 pg/mL. Prior to surgery, the patient was receiving optimized medical therapy consisting of phenprocoumon (dose adjusted according to INR values), telmisartan 20 mg once daily, bisoprolol 2.5 mg once in the morning and once in the evening, amiodarone 200 mg once daily, esomeprazole 40 mg once daily, and torasemide 10 mg once daily. Based on the clinical assessment and the patient’s underlying comorbidities, pre-operative medical treatment was considered optimized. In this case, MR appears to have two underlying mechanisms. Previous echocardiographic examinations had already demonstrated the presence of a leaflet perforation without signs of acute onset of severe MR. This finding is also consistent with the clinical history, as the patient did not report episodes of acute severe respiratory distress. Furthermore, pre-operative clinical examination revealed no peripheral oedema, no clinically relevant pleural effusion, and unremarkable lung auscultation findings. The leaflet perforation likely developed after the surgical procedure in 2009 and resulted in an eccentric regurgitant jet (see Supplementary material online, *Video S1*). In addition, the patient presents with atrial functional MR, characterized by annular dilatation, a central regurgitant jet, left atrial enlargement, and persistent atrial fibrillation. Over time, both regurgitant jets have shown progressive worsening (*Figure 3*). Quantitative echocardiographic assessment demonstrated an effective regurgitant orifice area of 0.4 and 0.3 cm^2^ proximal isovelocity surface area, a biplane vena contracta width of 13 mm, and a regurgitant volume of 70 mL, consistent with severe MR. The left ventricular end-diastolic dimension measured 5.2 cm and estimated pulmonary artery systolic pressure exceeded 50 mmHg. Cardiac CT further demonstrated annular dilatation with a mitral annulus area of 8.7 cm^2^, a perimeter of 109.1 mm, an inter-trigonal distance (TT) of 25.7 mm, an anterior-posterior diameter (AP) of 29.4 mm, and a septal-lateral diameter (SL) of 36.1 mm.

**Figure 3:**
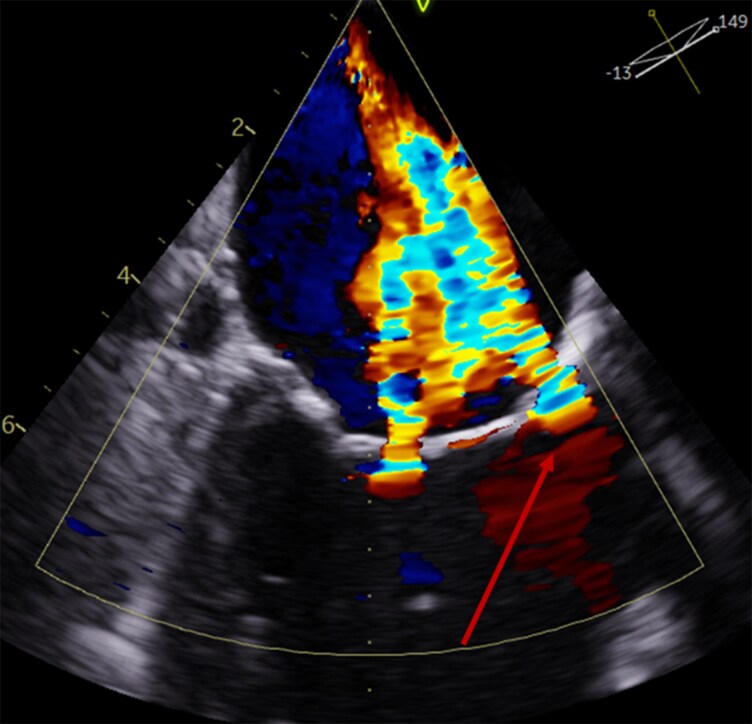
Transoesophageal echocardiography showing a serve mixed mitral regurgitation due to annular dilatation and leaflet perforation, with the arrow indicating a perforation secondary to patch dehiscence.

The case was reviewed by the Heart Team and M-TEER was considered unsuitable due to the leaflet perforation. Screening for the transseptal TMVI with the Sapien M3 prosthesis (Edwards Lifesciences, Irvine, USA) was negative due to an increased risk of paravalvular leakage with a likely requirement for an additional occluder device.

Computed tomography confirmed a favourable anatomical profile for implantation of the Tendyne prosthesis. The predicted neo-LVOT area measured 294.5 mm^2^ in end-systole, indicating a low risk of LVOT obstruction (*Figure 4*). In addition, no intracardiac thrombus was detected, the left ventricular ejection fraction was above 30%, and there was no evidence of severe mitral annular calcification. Based on these findings, the patient was accepted for intervention. Given the dual-frame design of the device, with its inner and outer frame, it was uncertain pre-procedurally whether the external frame would be able to provide sufficient coverage of the leaflet perforation.

**Figure 4:**
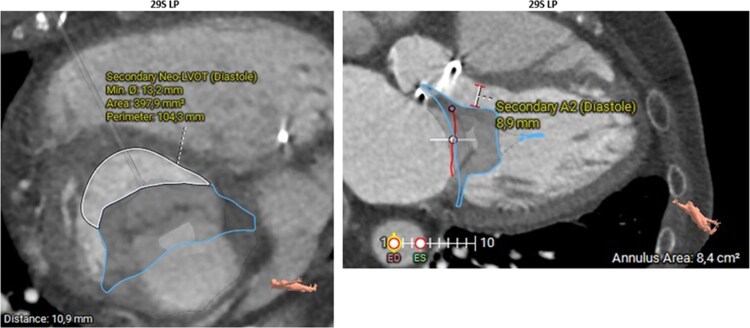
Computed tomography–based assessment of the Tendyne device in the presence of a mechanical aortic valve prosthesis, demonstrating a neo-left ventricular outflow tract area of 400 mm^2^.

A Tendyne™ 29 S LP valve was successfully implanted via a standard transapical approach under general anaesthesia. The device was positioned to ensure complete coverage of the leaflet perforation. Post-deployment transoesophageal echocardiography confirmed the complete elimination of MR, full sealing of the leaflet perforation by the *external stent frame*, absence of paravalvular leakage, unobstructed LVOT, and preserved mechanical aortic valve function (see Supplementary material online, *Video S2*). The post-interventional course was uneventful. The patient was discharged home on post-operative Day 7 in stable condition. At follow-up, she reported a marked improvement in functional status, corresponding to NYHA class I. Repeat echocardiography confirmed normal function of both the Tendyne mitral prosthesis and the mechanical aortic valve, with no recurrence of MR (*Figure 5*).

**Figure 5:**
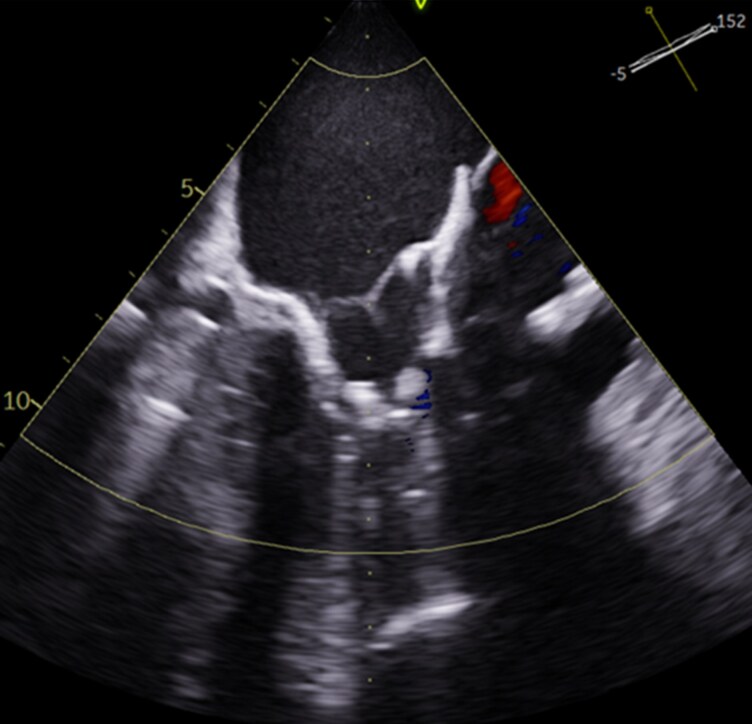
Transoesophageal echocardiography demonstrated the post-procedural result with no residual mitral regurgitation.

## Discussion

This case illustrates the feasibility and effectiveness of the Tendyne® TMVI system in a patient with complex post-surgical mitral valve disease and prior mechanical aortic valve replacement. The unique dual-frame design of the Tendyne prosthesis, incorporating an atrial cuff and an external stent frame, enabled complete sealing of a leaflet perforation caused by patch dehiscence, thereby avoiding the need for additional closure devices. Importantly, the prosthesis was implanted without mechanical or haemodynamic interference with the existing mechanical aortic valve, confirming the technical compatibility of the two devices.

These findings are consistent with the feasibility study by Taramasso *et al*., who reported complete MR elimination and no interaction between the Tendyne valve and pre-existing aortic prostheses in patients with prior surgical or transcatheter aortic valve replacement.^5^ The decision-making process in this case highlights the critical role of a multidisciplinary Heart Team. Detailed anatomical assessment using advanced imaging enabled precise selection of the device and approach, tailored to the patient’s specific post-surgical anatomy.

Following comprehensive evaluation of all clinical and anatomical factors, the Heart Team should also carefully consider whether a patient is likely to benefit more from achieving a greater reduction in MR or from minimizing procedural risk. Recent comparative analyses indicate that TMVI can provide a more complete and durable elimination of MR compared with TEER, although at the cost of a higher incidence of peri-procedural complications.^2^ Therefore, treatment strategies must be individualized, balancing efficacy and safety according to each patient’s anatomy, comorbidities, and overall risk profile.

Alternative transcatheter options, such as transseptal TMVI with the Sapien M3 system, were deemed unsuitable in this case due to the high likelihood of residual regurgitation and the potential need for additional occlusion devices. Data from the Tendyne Expanded Clinical Study further support the safety and efficacy of the system, demonstrating consistent MR elimination, absence of intra-procedural mortality, and significant improvements in symptoms and quality of life.^6^

In conclusion, transapical TMVI with the Tendyne prosthesis represents a safe and effective therapeutic option for selected high-risk patients with severe MR and complex post-surgical anatomy, including leaflet perforations or paravalvular leakage. To our knowledge, this is the first reported case in which the external stent of the Tendyne prosthesis successfully sealed a mitral leaflet perforation. This emphasizes the potential advantages of the Tendyne design in addressing complex structural defects and the importance of imaging-guided, multidisciplinary planning in optimizing procedural outcomes.

## Supplementary Material

ytag441_Supplementary_Data

## Data Availability

The data underlying this article will be shared on reasonable request to the corresponding author.

## Lead author biography

V. Trenkpohl is a cardiology resident at the Heart and Diabetes Center NRW in Germany and is currently in her third year of specialist training. She completed her medical studies at Saarland University in Homburg and successfully obtained her doctoral degree through an experimental research project in neurosurgery. Her scientific and clinical interests focus on interventional cardiology, including catheter-based therapies and structural heart disease. Dr Trenkpohl is an active member of the European Society of Cardiology (ESC) and the German Society of Cardiology (DGK).

## Notes

**Consent:** Patient consented to an anonymized publication according to the COPE guidelines.
